# Atypical Cellular Elements of Unknown Origin in the Subbasal Nerve Plexus of a Diabetic Cornea Diagnosed by Large-Area Confocal Laser Scanning Microscopy

**DOI:** 10.3390/diagnostics11020154

**Published:** 2021-01-21

**Authors:** Katharina A. Sterenczak, Oliver Stachs, Carl Marfurt, Aleksandra Matuszewska-Iwanicka, Bernd Stratmann, Karsten Sperlich, Rudolf F. Guthoff, Hans-Joachim Hettlich, Stephan Allgeier, Thomas Stahnke

**Affiliations:** 1 Department of Ophthalmology, Rostock University Medical Center, 18057 Rostock, Germany; oliver.stachs@uni-rostock.de (O.S.); karsten.sperlich@uni-rostock.de (K.S.); rudolf.guthoff@med.uni-rostock.de (R.F.G.); thomas.stahnke@med.uni-rostock.de (T.S.); 2 Department Life, Light & Matter, University of Rostock, 18059 Rostock, Germany; 3 Department of Anatomy, Cell Biology & Physiology, Indiana University School of Medicine-Northwest, Gary, Indianapolis, IN 46204, USA; cmarfurt@iun.edu; 4 Eye Clinic, Johannes Wesling Klinikum Minden, Ruhr-University Bochum, 32429 Minden, Germany; aleksandra.matuszewska@gmail.com (A.M.-I.); hettlich@t-online.de (H.-J.H.); 5 Heart and Diabetes Center NRW, Ruhr-University Bochum, 32545 Bad Oeynhausen, Germany; bstratmann@hdz-nrw.de; 6 Institute for Automation and Applied Informatics, Karlsruhe Institute of Technology, 76344 Eggenstein-Leopoldshafen, Germany; stephan.allgeier@kit.edu

**Keywords:** in vivo large-area confocal laser scanning microscopy, subbasal nerve plexus, keratocyte-derived myofibroblasts, Schwann cells, diabetes

## Abstract

In vivo large-area confocal laser scanning microscopy (CLSM) of the human eye using EyeGuidance technology allows a large-scale morphometric assessment of the corneal subbasal nerve plexus (SNP). Here, the SNP of a patient suffering from diabetes and associated late complications was analyzed. The SNP contained multiple clusters of large hyperintense, stellate-shaped, cellular-like structures. Comparable structures were not observed in control corneas from healthy volunteers. Two hypotheses regarding the origin of these atypical structures are proposed. First, these structures might be keratocyte-derived myofibroblasts that entered the epithelium from the underlying stroma through breaks in Bowman’s layer. Second, these structures could be proliferating Schwann cells that entered the epithelium in association with subbasal nerves. The nature and pathophysiological significance of these atypical cellular structures, and whether they are a direct consequence of the patient’s diabetic neuropathy/or a non-specific secondary effect of associated inflammatory processes, are unknown.

**In vivo confocal laser scanning microscopy of the cornea.** The cornea represents a biological barrier to and mediator of the external environment and consists of five distinct layers: The epithelium, including the subbasal nerve plexus (SNP); Bowman’s layer; stroma; Descemet’s membrane; and the endothelium [1]. Each of these layers fulfills specific biological functions which are crucial to ocular homeostasis. In addition, the cornea is highly innervated by sensory nerves that exert important influences on the regulation of corneal epithelial integrity and wound healing [2]. Corneal nerves lose their myelin sheathes soon after entering the cornea at the limbus and become essentially transparent; thus, their clinical appearance was not amenable to in vivo evaluation until the development of confocal laser scanning microscopy (CLSM) [3]. During in vivo CLSM, corneal elements, including cells, the extracellular matrix, and nerves, scatter light at various degrees, making it possible to obtain high signal-to-noise images leading to high contrast microscopic imaging of the native cornea at the cellular level [4]. Several studies have shown that SNP changes are not characteristic of one specific corneal pathology, but reflect non-specific pathological processes which are present in many corneal, ocular, or systemic diseases [5,6] or arise as a result of a therapy regime, such as that used to treat multiple myeloma [7]. The in vivo CLSM scans of the SNP within this study were performed by using Heidelberg Retina Tomograph II (HRT II, Heidelberg Engineering GmbH, Heidelberg, Germany) in combination with an in-house modified version of the Rostock Cornea Module (RCM, Heidelberg Engineering GmbH, Heidelberg, Germany)—the RCM 2.0—and the EyeGuidance system, which enabled large-scale imaging of the SNP [8,9,10]. During conventional CLSM, single images represent a standard area of 0.16 mm^2^ (Figure 1, inset B), thereby covering approximately 0.2% of the average complete corneal surface, which is insufficient for conducting a reliable morphometric assessment of the complete SNP [5]. In the past, mosaicking approaches of single images have been proposed in order to examine the SNP on a larger scale. The EyeGuidance system applied in this study represents a highly automated computer-controlled technique that facilitates the generation of mosaic images by using a moving fixation target which is presented to the contralateral eye [8,9]. Figure 1 and Figure S1 display a normal SNP of a healthy volunteer using large-area CLSM.

**Patient’s history**. The patient presented here has suffered from type 2 diabetes mellitus (DM) since 2005, with poorly adjusted blood-sugar control (HbA1c 9.5%). Further key diagnoses include bilateral diabetic nephropathy (actual grade II), diabetic polyneuropathy, steatosis hepatitis, coronary vascular disease, arteriosclerosis, sleep apnea syndrome, adipositas grade III, and hyperlipidemia. Specific ophthalmological diagnoses include mild non-proliferative retinopathy (NPDR) and a mild epiretinal gliosis which was detected by optical coherence tomography (OCT). Slit-lamp examination showed slight subepithelial densities within the anterior stroma of the cornea. In addition to the standard ophthalmological diagnostic methods, we examined the patient’s eye using CLSM with large-area imaging techniques [8,9]. 

**Findings in the patient’s SNP by large-area CLSM**. The SNP of the patient (Figure 2 and Figure S2) contained a large number of atypical hyperintense, cellular-like structures to an extent that has never been observed before by us or described in the literature. Abnormalities in the corneal epithelium are one of the most common and long-term complications of DM [5,11]. Other DM patients (pictures not shown here) with hyperglycemia have also shown similar structures in their SNPs, but not to the same extent as the patient described here. Chronic hyperglycemia has been reported to trigger the expression of various cytokines, chemokines, and cell adhesion molecules, resulting in their over-expression and contribution to the development of ocular complications in DM [11]. Based on this, we hypothesize that the unusual cellular structures detected in the patient’s SNP could reflect abnormal cellular activities within the basal epithelium caused by inflammatory processes. Regarding the origin of these atypical cellular elements, we would like to propose the following two hypotheses (Hypothesis 1 and 2).

**Hypothesis** **1.** *The Cellular-Like Elements are Keratocyte-Derived Myofibroblasts*.

Jester et al. [12] investigated the light-scattering and actin organization of rabbit keratocytes under various culture conditions using confocal reflectance microscopy and fluorescence. Rabbit keratocytes cultured in serum-free conditions showed a characteristic dendritic morphology with predominantly cortical actin organization [12]. Fibroblastic keratocytes stimulated with platelet-derived growth factor (PDGF) appeared elongated and spindle-shaped with prominent intracellular actin filament bundles; fibroblasts stimulated with fibroblastic growth factor (FGF-2) appeared broader and flatter; and myofibroblasts cultured with transforming growth factor beta (TGF-β) showed a large, spreading morphology with extensive intracellular stress fibers [12]. Of interest, PDGF has been reported to affect the processes of DM and its complications, largely via diverse signaling pathways, such as reactive oxygen species (ROS) [13]. A comparison of these in vitro observations and our in vivo findings detected numerous similarities in size and shape between cultured keratocytes/myofibroblasts and our detected hyperintense structures. 

According to Wilson’s et al. [14] paradigm of fibrosis, the epithelial basement membrane (EBM) is intact during corneal homeostasis and blocks epithelial TGF-β and PDGF from penetrating into the underlying stroma. After epithelial injuries, the epithelium and the EBM are disrupted and TGF-β and PDGF are activated and penetrate the underlying stroma, driving the development of myofibroblasts from keratocyte-derived and bone-marrow-derived (fibrocyte) precursors [14]. Myofibroblasts themselves secrete disordered collagen type 1, collagen type 3, and other matrix material that disrupt the normal stromal lamellae to produce corneal opacity or scarring [14]. After severe injuries of the cornea where the basement membrane is damaged, large numbers of myofibroblasts are generated, which persist in the corneal stroma and secrete disorganized extracellular matrix components, leading to fibrosis and alterations in the structure and function of the corneal stroma [15]. 

Morphological changes in Bowman’s layer are associated with advanced bullous keratopathy and Fuchs’s corneal dystrophy [16]. During disease progression, the epithelium becomes increasingly dysfunctional and at late stages, changes also occur in Bowman’s layer [16]. According to one hypothesis [16], at some point, keratocytes or their progeny, corneal fibroblasts, and myofibroblasts are no longer confined to the underlying stroma, but enter Bowman’s layer. Corneal fibroblasts eventually advance to the immediate subepithelial region and differentiate into alpha-smooth muscle actin (α-SMA)-expressing myofibroblasts. These histopathological changes noted in bullous keratopathy or Fuchs’ dystrophy support the hypothesis that Bowman’s layer is actively maintained during an individual’s life by regulatory systems that are compromised by epithelial dysfunction associated with the progression of these diseases [16]. Cytokines, growth factors, or other modulators that comprise this regulatory system likely modulate the keratocyte phenotype, localization, and viability [16]. In a case report by Shetty et al. [17], breaks in Bowman’s layer and SNP changes were observed in a Dry Eye patient with chronic pain and Vitamin D deficiency. These authors proposed that ocular surface inflammation leads to increased epithelial permeability and rarefaction of basal epithelial cells. Changes of subbasal nerves coupled with epithelial changes lead to microscopic breaks in Bowman’s layer, providing a conduit for inflammatory factors to enter the stroma [17]. Changes in the subbasal nerves may also trigger the release of proteolytic enzymes that can lead to further enlargement of the breaks propagating more inflammation [17]. 

In summary, the mechanisms described above could also have led to the migration of stromal cellular elements into the epithelium of our patient. Due to continuous inflammatory processes affecting the epithelial basement membrane and subbasal nerves, keratocyte-derived myofibroblasts from the stroma could have entered Bowman’s layer through breaks. It is of interest to note in our patient that not all breaks in Bowman’s layer are associated with abnormal cells, and conversely, not all abnormal cells are located near visible breaks in Bowman’s layer. Concerning the latter observation, it is tempting to speculate that the formation of breaks in Bowman’s layer is a dynamic and transient occurrence, i.e., a break forms, stromal cells migrate through the opening into the epithelium, and the break then closes, leaving no evidence of its prior existence upon CLSM examination.

**Hypothesis** **2.** *The Cellular-Like Elements are Schwann Cells*.

It is well-known that corneal nerves originate from the trigeminal ganglion and enter the corneal stroma at the corneal limbus [18,19,20,21]. The nerve bundles lose their perineurium and myelin sheaths within approximately 1 mm of the limbus and continue into the corneal stroma only surrounded by non-myelinating Schwann cell sheaths forming “Remak bundles” [22]. These nerve bundles subdivide repeatedly to form smaller nerve branches that enter the superficial stroma located just beneath Bowman’s layer [20,21]. Within the corneal stroma, keratocytes are often found in proximity to the nerve fibers and occasionally enwrap adjacent nerve fibers in cytoplasmic extensions [20,21]. As Schwann cells are the primary glial cells of the peripheral nervous system (PNS), they also have several additional functions in the healthy cornea [23]. Along with the insulating properties of the axons as a prerequisite for stimulus transmission, they possess phagocytotic properties and remove axonal debris after injury and coordinate cytokine signaling and inflammatory responses with macrophages. In order to accomplish these functions, Schwann cell dedifferentiation, proliferation, migration, and re-differentiation processes are necessary [24]. Stromal nerves enwrapped by Schwann cells penetrate Bowman’s layer to provide sensitivity to the ocular surface. In humans, approximately 400–500 widely spaced stromal nerves penetrate Bowman’s layer, mainly in the peripheral and intermediate cornea, to produce the epithelial subbasal nerve plexus [18]. The subbasal nerves lose their Schwann cell wrappings immediately upon entering the basal epithelium. It is known that corticosteroids regulate inflammation and cell proliferation and can be synthesized by peripheral nerves [25]. Schwann cells are very sensitive to these endogenous corticosteroids and can react with changes in morphology and proliferation [25]. The morphological appearance of the atypical cellular elements seen in our patient is reminiscent of glial cells and suggests that a potential imbalance of existing cytokines and hormones could have caused an uncontrolled migration and proliferation of Schwann cells in some central areas of the patient’s cornea. 

It should also be noted that mild epiretinal gliosis was detected by optical coherence tomography (OCT) in our patient. Epiretinal gliosis is associated with changes in cytokine secretion driven by macroglia. It has been shown by Eastlake and colleagues [26] that the majority of cytokines and inflammatory factors were produced by Müller glia cells and included the granulocyte colony stimulating factor (G-CSF), monocyte chemoattractant protein-1 (MCP-1), platelet-derived growth factor BB (PDGF-bb), CC-chemokine ligand 5 (also known as RANTES), vascular endothelial growth factor (VEGF), and transforming growth factor-beta 2 (TGF-β2). This process is called reactive gliosis, through which glial cells seek to maintain retinal homeostasis. When malfunctioning, macroglial cells can become primary pathogenic elements. Reactive gliosis has been described in different retinal pathologies, including age-related macular degeneration, diabetes, glaucoma, retinal detachment, and retinitis pigmentosa [27]. We hypothesize that the cytokines involved in these processes can also enter the anterior segment of the eye and the aqueous humor via diffusion through the vitreous, where they may ultimately reach the cornea and activate Schwann cells. 

Lastly, it must be acknowledged that our hypotheses are only based on *in vivo* morphological data. The correlation between in vivo morphology and the real identity and composition of the findings could only be determined by a comparison with other *ex vivo* methods, such as immunohistology, which was not practicable here. 

In conclusion, the SNP of the patient presented in this paper contained extensive patches of large, stellate-like cellular elements of unknown origin. Based on the patient’s history and the characteristic morphological appearance of the cellular elements, two hypotheses are proposed. The first hypothesis is that these elements are keratocyte-derived myofibroblasts from the underlying stroma that have entered the epithelium through transient breaks in Bowman’s layer caused by inflammatory processes. The second hypothesis is that these elements are Schwann cells that have infiltrated the epithelium in association with corneal subbasal nerves. The morphological appearance of the cells is consistent with either hypothesis. The atypical cellular elements within the patient’s SNP could have been caused by his DM and chronic hyperglycemia or, more likely, they may have developed as a non-specific secondary effect of associated inflammatory processes. This study reflects the clinical diagnostic potential of the in vivo large-area CLSM technique in terms of detecting early changes and investigating the longitudinal effects of diseases or treatment regimens affecting the SNP.

## Figures and Tables

**Figure 1:**
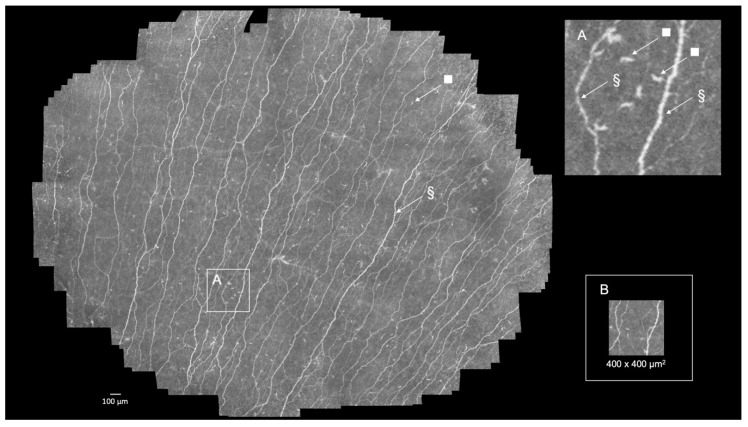
Normal subbasal nerve plexus (SNP) of a healthy volunteer obtained from a 55-year-old male using large-area confocal laser scanning microscopy (CLSM). Numerous hyperintense subbasal nerves of varying diameters, many of which are associated with dendritic cells, are visible (§, nerves of the SNP, and □, dendritic cell) (high-resolution image supplementary Figure S1). Inset (**A**): subbasal nerve (§) and dendritic cell (□), and inset (**B**): single CLSM image (area 400 × 400 µm^2^).

**Figure 2:**
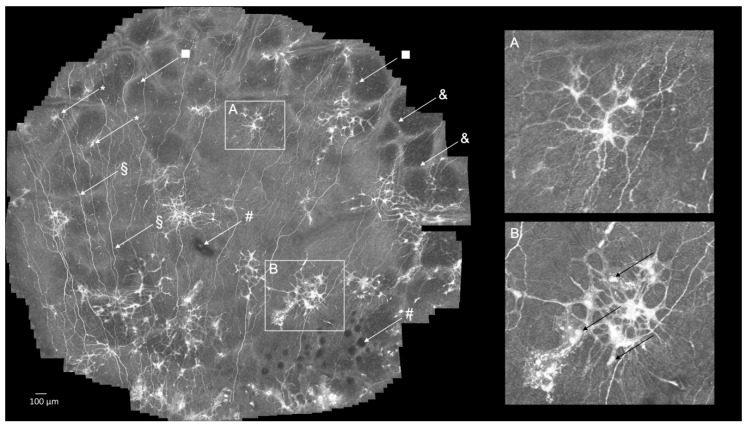
Subbasal nerve plexus (SNP) in the cornea of a 71-year-old male admitted to hospital due to hyperglycemia. Clusters of hyperintense, large, cellular-like structures are present throughout the central cornea (§, nerves of the SNP; &, applanation artifacts; #, hypointense areas of unknown origin; *, neuroma; and □, dendritic cell) (high-resolution image supplementary Figure S2). Inset (**A**): the cellular-like structures are stellate-shaped and appear to possess multiple cytoplasmic processes, and inset (**B**): hyperintense cellular structures with enclosed “granular-like” accumulations which could represent cell nuclei (black arrow).

## Data Availability

The data presented in this study are available on request from the corresponding author.

## Supplementary Materials

The following are available online at https://www.mdpi.com/2075-4418/11/2/154/s1.
