# Supplementary material for: Atypical Cellular Elements of Unknown Origin in the Subbasal Nerve Plexus of a Diabetic Cornea Diagnosed by Large-Area Confocal Laser Scanning Microscopy
Source: Diagnostics (Basel). 2021 Jan 21;11(2):154. doi: 10.3390/diagnostics11020154 (PMC7911241; doi:10.3390/diagnostics11020154)
Supplement: Supplementary file 1 [file diagnostics-11-00154-s001.pdf]

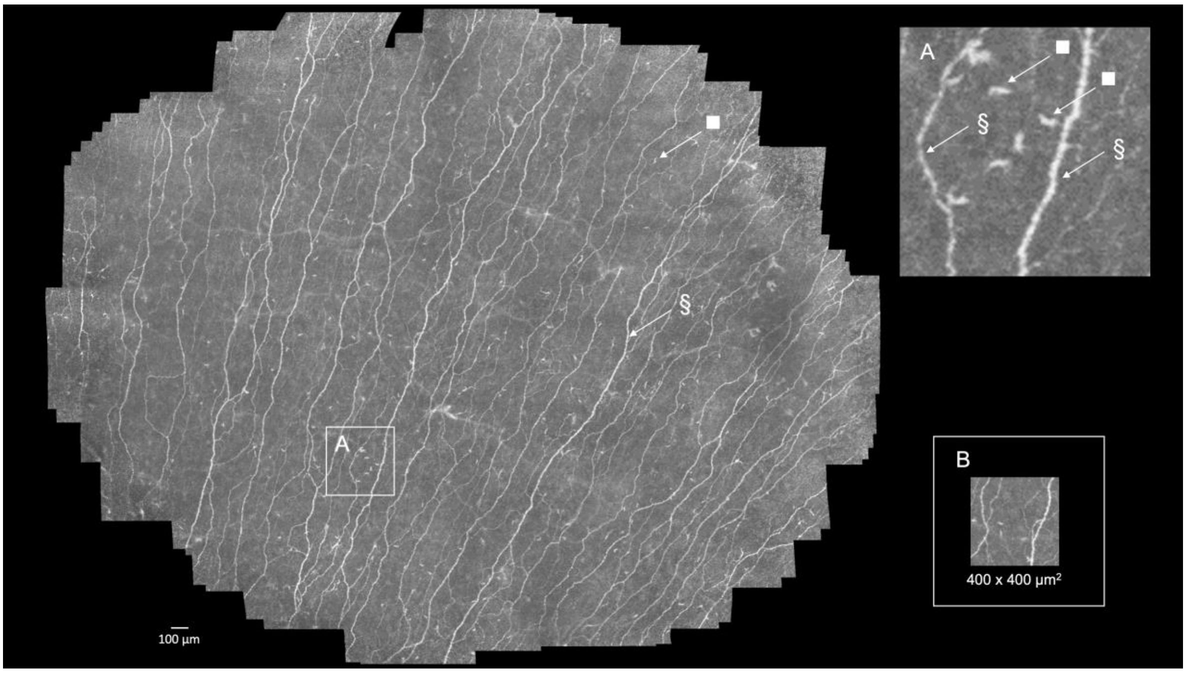

Figure S1. High-resolution image of Figure 1. Normal subbasal nerve plexus (SNP) of a healthy volunteer using large-area confocal laser scanning microscopy (CLSM). Numerous hyperintense subbasal nerves of varying diameters, many of which are associated with dendritic cells, are visible (§, nerves of the SNP, and □, dendritic cell). Inset (**A**): subbasal nerve (§) and dendritic cell (□), and inset (**B**): single CLSM image (area 400 × 400 μm²).

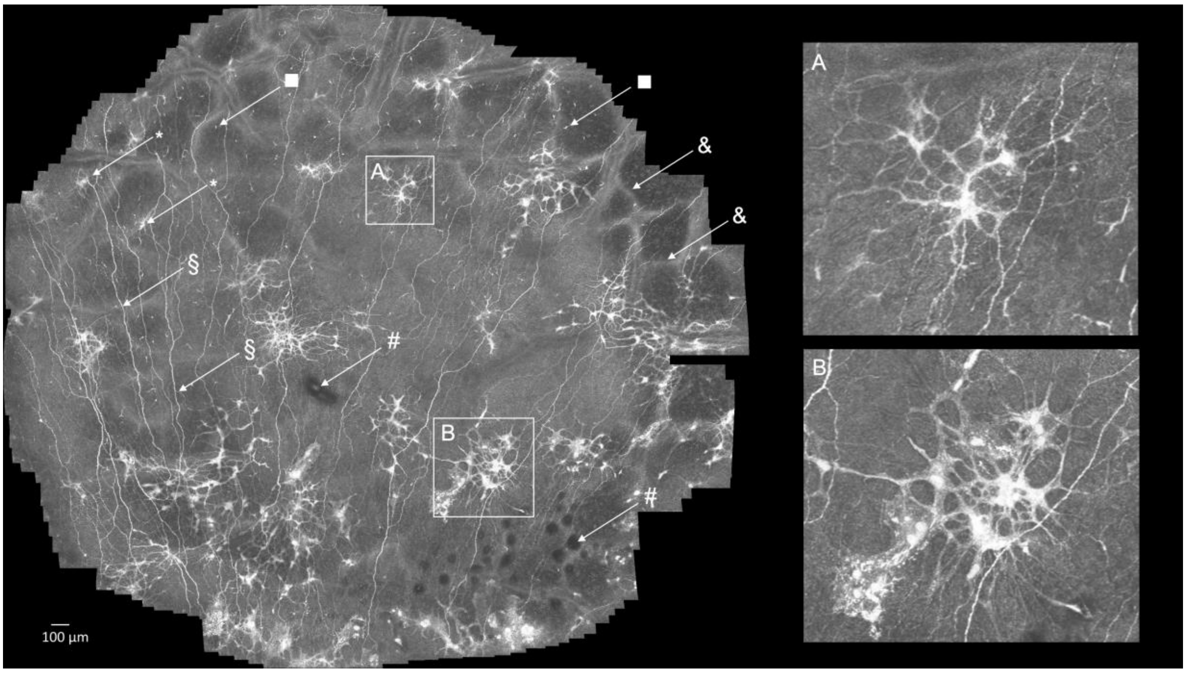

Figure S2. High-resolution image of Figure 2. Patient's subbasal nerve plexus (SNP) using large-area confocal laser scanning microscopy (CLSM). Clusters of hyperintense, large, cellular-like structures are present throughout the central cornea (§, nerves of the SNP; &, applanation artifacts; #, hypointense areas of unknown origin; *, neuroma; and □, dendritic cell). Inset (**A**): the

cellular-like structures are stellate-shaped and appear to possess multiple cytoplasmic processes, and inset (**B**): hyperintense cellular structures with enclosed "granular-like" accumulations which could represent cell nuclei (black arrow).
